# Editorial: Role of stem cell derivatives in inflammatory diseases

**DOI:** 10.3389/fimmu.2023.1275068

**Published:** 2023-08-31

**Authors:** Cuiping Zhang, Zhe Li, Jianghuai Wang, Yongming Yao, Haihong Li, Xiaobing Fu

**Affiliations:** ^1^ Research Center for Tissue Repair and Regeneration affiliated to the Medical Innovation Research Department of PLA General Hospital, Beijing, China; ^2^ Research Unit of Trauma Care, Tissue Repair and Regeneration, Chinese Academy of Medical Sciences, Beijing, China; ^3^ Innovation Center for Wound Repair, West China Hospital, Sichuan University, Chengdu, Sichuan, China; ^4^ Burns Unit, Concord Hospital, University of Sydney Medical School, Sydney, Australia; ^5^ National University of Ireland (NUI), Cork University Hospital, Cork, Ireland; ^6^ Translational Medicine Research Center, The Center of Translational Medicine affiliated to the Medical Innovation Research Department of PLA General Hospital, Beijing, China; ^7^ Department of Burns and Plastic Surgery, The Seventh Affiliated Hospital of Sun Yat-sen University, Shenzhen, Guangdong, China

**Keywords:** stem cells, derivatives, inflammatory diseases, treatment, immune

Inflammation is a complex and protective response of the immune system to pathogens. Appropriate inflammation response is beneficial to pathogen elimination and tissue repair, but uncontrolled inflammatory reactions often result in the damage of normal tissues (1). In recent years, there are more and more people troubled with inflammatory diseases including chronic wounds, asthma, osteoarthritis, and sepsis (2). The intractable inflammatory diseases were caused by disorders of immune systems, imposing a heavy burden on society and individuals. Innate and adaptive immune cells, such as neutrophils, macrophages, and lymphocytes, as well as their secreted cytokines contribute to the pathogenesis of inflammatory diseases. Recent research advances have enhanced our comprehension of the basic pathogenesis of the most common inflammatory diseases, which bring about the development of some innovative approaches.

In the past decades, stem cell therapy has been used as a potential therapeutic intervention for various human diseases due to their special characteristics. For example, mesenchymal stem cells (MSCs) are thought to be the most sought-after stem cells with immunomodulatory property for treating a variety of inflammatory diseases (3). However, safety concerns have limited the clinical application of stem cells. It is indispensable to promoting the development of safe and efficient therapeutic strategies based on stem cells. Recently, the performance of stem cells after transplantation has increasingly been attributed to their exocrine function. It is evident from the literature that stem cell derivatives including extracellular vesicles (EVs) can mimic the function of stem cells without the concerns of immune response and ethical issues (4).

EVs contain diverse cellular molecules including RNAs, DNAs, and proteins, promoting the information exchange between cells. Compared with their parental stem cells, EVs have several advantages including high safety, absence of immune reactions, fewer ethical issues, and decreased potential for embolism formation and carcinogenicity. Recent research reported the pivotal role of stem cell-derived EVs (SC-EVs) in a wide spectrum of diseases such as cancer, myocardial infarction, and skin wounds (5–7). But the study on the role of SC-EVs in inflammatory diseases is still in the infancy.

The Research Topic of “*The Role of* Stem Cell Derivatives *in Inflammatory Diseases*” was designed to introduce the role of stem cell derivatives, especially EVs, in the pathogenesis, diagnosis, and treatment of inflammatory diseases. A total of 12 manuscripts were accepted in the topic, including 4 original articles, 7 reviews, 1 mini review, 8 from China. 2 from United States, 1 from Poland, and 1 from Vietnam.

Skin wound healing is a highly sophisticated process consisting of four distinct and overlapping phases: hemostasis, inflammation, proliferation, and remodeling (8). Chronic wounds usually arise owning to halt at one or more points in above phases, especially in inflammation phase. Wei et al. reviewed the effects of MSC-EVs on the function of skin repair cells including inflammatory cells, vascular endothelial cells, fibroblasts, and epidermal cells and application avenues of MSC-EVs on wounds such as local injection and combination with biomaterials. Jia et al. focused on the potential of EVs derived from adipose-derived stem cells (ADSC-EVs) in regulating wound inflammation and discuss the mechanisms underneath this phenomenon. Atopic dermatitis (AD) and psoriasis are systemic and immune-allergic inflammatory skin diseases (9). The review article by Yang et al. brought an extensive overview of the therapeutic effects of MSCs and their derivatives including EVs on AD and psoriasis.

Four original researches in this topic provide strong evidence for the therapeutic effects of SC-EVs on inflammatory diseases. Acute radiation syndrome (ARS) is associated with the exposure to high doses of radiation and featured by immune suppression and organ failure. Seim et al. identified EVs from the plasma of mice after whole body irradiation (WBIR) as important participants in ARS and for the first time demonstrated that MSC-EV administration prior to WBIR would decrease ARS. Asthma is a chronic respiratory disease featured by airway inflammation and remodeling. Xu et al. identified that Hypoxic EVs derived from human umbilical cord MSCs (Hypo-hUCMSC-EVs) can reduce allergic airway inflammation and remodeling by atomizing inhalation. Additionally, for treatment of osteoarthritis and chondrocyte-related disorders, Nguyen et al. investigated the effects of EVs released from hUCMSC primed by cytokines including transforming growth factor beta (TGFβ), interferon alpha (IFNα), or tumor necrosis factor alpha (TNFα) on osteoarthritic chondrocyte physiology. In an ovine pneumonia/sepsis model, Homma et al. reported the beneficial effects of MSCs (10×10^6^ cells/kg) isolated from bone marrow (BM- MSCs) on sepsis-induced multiorgan dysfunctions, but EVs derived from the same amount of BM-MSCs failed to function. The possible reason may attribute to the small dose of the EVs and repeated treatment should be performed.

Treatments with SC-EVs for other inflammatory diseases such as periodontitis and inflammation-induced fibrosis were also reviewed by Cai et al. and Lv et al. The review written by Zhao et al. summarized the current researches on mechanism of MSC-EVs affecting inflammatory diseases by modulating epigenetic modification. Liu et al. and Karnas et al. provided reviews of immunomodulatory effects of SC-EVs on immune system and immune cells including macrophages, granulocytes, mast cells, natural killer cells, dendritic cells, T cells, and B cells in some inflammatory disease models.

These articles present studies in the treatment of inflammatory diseases with EVs derived from stem cells of various sources. Based on the given results, SC-EVs are suggested as a potential substitute for stem cell therapy. So, this topic provides a critical guide to clinic application of SC-EVs for the treatment of inflammatory diseases.

## Author contributions

CZ: Writing – review & editing, Writing – original draft. ZL: Writing – review & editing. JW: Writing – review & editing, Validation. YY: Writing – review & editing, Writing – original draft. HL: Writing – original draft, Writing – review & editing. XF: Writing – original draft, Writing – review & editing.
